# Dynamics of HSD17B3 expression in human fetal testis: implications for the role of Sertoli cells in fetal testosterone biosynthesis

**DOI:** 10.3389/fcell.2024.1429292

**Published:** 2024-07-30

**Authors:** Ana Planinic, Tihana Maric, Marta Himelreich Peric, Davor Jezek, Ana Katusic Bojanac

**Affiliations:** ^1^ Department of Histology and Embryology, University of Zagreb School of Medicine, Zagreb, Croatia; ^2^ Scientific Centre of Excellence for Reproductive and Regenerative Medicine, University of Zagreb School of Medicine, Zagreb, Croatia; ^3^ Department of Medical Biology, University of Zagreb School of Medicine, Zagreb, Croatia

**Keywords:** testosterone, Sertoli, testis, human, fetal, Leydig

## Abstract

**Introduction:** Androgens play a pivotal role in shaping male sexual characteristics, with testosterone being an essential hormone in orchestrating various developmental processes. Testosterone biosynthesis involves a series of enzymatic reactions, among which the 17β-hydroxysteroid dehydrogenase type 3 (HSD17B3) holds significance. While its role in adult Leydig cells is well established, its localization and importance during the fetal period remain less known, especially in humans. This study aims to delineate the dynamics of HSD17B3 expression in human fetal testes to clarify the contribution of specific cell types to testosterone biosynthesis.

**Methods:** Using immunofluorescence staining, we investigated the expression pattern of HSD17B3 in human fetal and adult testicular tissues.

**Results and discussion:** The findings of this study revealed a distinct temporal and cellular expression pattern of HSD17B3 protein in the fetal period. We detected its expression exclusively in Sertoli cells, the highest during the second trimester. This unique localization suggests the inclusion of fetal Sertoli cells in testosterone production during the critical masculinization-programming window. Furthermore, we demonstrated a shift in HSD17B3 expression from Sertoli cells to Leydig cells in adulthood, corroborating findings from rodent studies. This study sheds light on the intricate, still underexplored regulation of steroidogenesis during fetal development, whose disturbance might lead to testicular dysgenesis. Further research is warranted to elucidate the regulatory pathways governing the expression of HSD17B3 and its transition between Sertoli and Leydig cells, potentially paving the way for novel therapeutic interventions in disorders of sexual development.

## 1 Introduction

Androgens are steroid hormones that regulate the development and maintenance of the male sexual phenotype. The primary androgen in males is testosterone, but the more potent dihydrotestosterone (DHT) and less potent androstenedione and dehydroepiandrosterone (DHEA) also have important roles (Roy et al., 1999). Testosterone has anabolic effects on male musculoskeletal development as well as androgenic effects such as spermatogenesis and fertility, voice deepening, sebum production, sex drive, and erection (Chang, 2002). The majority of testosterone in adults is produced by Leydig cells through steroidogenesis. The classical testosterone production pathway uses cholesterol as a precursor molecule, mobilized by the steroidogenic acute regulatory (STAR) protein from lipid droplets or the cell membrane into mitochondria. The enzyme cytochrome P450 family 11 subfamily A member 1 (CYP11A1) then converts cholesterol into pregnenolone, which is further converted into DHEA by the cytochrome P450 family 17 subfamily A member 1 (CYP17A1) enzyme in the smooth endoplasmic reticulum. DHEA is then converted to androstenediol by HSD17B3, which is then converted into testosterone by HSD3B2 and secreted into the blood (Payne and Hardy, 2007). Circulating testosterone is further converted in peripheral tissues such as the prostate, skin, liver, and hair follicles by 5α-reductase (SRD5A) into dihydrotestosterone (DHT), which exhibits a more potent agonistic effect on the androgen receptor (Chang, 2002). Although circulating testosterone is the primary source for the majority of intracellular DHT production (Okon et al., 1980), the latter can also be produced from androsterone via a so-called backdoor biochemical pathway, which includes other enzymes, such as aldo-keto reductase family 1 member C2 and C4 (AKR1C2, AKR1C4), and 17β-hydroxysteroid dehydrogenase 6 (HSD17B6) (Swerdloff et al., 2017).

HSD17B3 is a member of the 17β-hydroxysteroid dehydrogenase (HSD17B) family, a group of enzymes that catalyze the last step in the synthesis of androgens, a conversion of androstenedione to testosterone (Labrie et al., 1997). This superfamily of enzymes consists of 15 isoforms, which are expressed in various tissues and have multiple functions (Wang et al., 2016; Hilborn et al., 2017). For example, the HSD17B3 isoform is expressed in the Leydig cells of the adult testis, while HSD17B5 (AKR1C3) is expressed in other tissues such as the adrenal gland and the ovarian thecal cells, thus referred to as the peripheral HSD17B (Melmed, 2016; Penning, 2019). HSD17B3 deficiency causes male pseudohermaphroditism, leading to ambiguous or female external genitalia, cryptorchidism, and infertility (Longo et al., 1975). Affected individuals are usually raised as females, and the diagnosis is made at puberty when they start to show virilization and have a high androstenedione/testosterone ratio (Furtado et al., 2012). Virilization is caused either by an abnormal enzyme that produces enough testosterone or by peripheral conversion of androstenedione to testosterone via HSD17B5 (Faienza et al., 2020). Patients with HSD17B3 deficiency exhibit the highest rates of gender dysphoria, with incidence up to 63%, similar to those with SRD5A deficiency (Furtado et al., 2012). Moreover, mutations of AKR1C genes involved in the backdoor pathway also appear to cause a similar pathology, pointing to the crucial role of testosterone and DHT in human gonadal development (Fluck et al., 2011; Furtado et al., 2012).

In rodents, fetal LCs were shown to have low CYP11A1, HSD3B1, and CYP17A1 levels in the cytoplasm, allowing them to produce androgens such as androstenedione and androsterone. However, it has been discovered that, in rat and mouse fetuses, HSD17B3 is not expressed in Leydig cells but, surprisingly, in Sertoli cells, implicating them as testosterone producers during gestation in rodents (Shima et al., 2013). Even 10 days after birth, HSD17B3 expression is still limited to Sertoli cells, but later, at day 30, which corresponds to the puberty period in humans, it is switched to Leydig cells (O’Shaughnessy et al., 2000). Importantly, HSD17B3 KO male mice exhibit normal masculinization and fertility, indicating that HSD17B3 is probably important for maximal but not basal testosterone production in the mouse testis (Rebourcet et al., 2020).

Recent studies on transcriptomes of human fetuses showed that HSD17B3 mRNA was not found in human fetal Leydig cells but in the Sertoli cell lineage (Guo et al., 2021), thus supporting the results from rodent studies. However, due to a lack of human fetal samples, no studies have confirmed this exclusivity in testicular tissues at the protein level. Hence, this study aimed to prove the abovementioned observation and showed for the first time the dynamics of HSD17B3 protein expression during various stages of human development, implying that Sertoli cells are local testosterone producers in fetuses.

## 2 Materials and methods

### 2.1 Sample collection and ethical approval

Human fetal (gestational age 14–33) paraffin-embedded testicular tissue (n = 16) from stillbirths was acquired from the archive of the Department of Histology and Embryology, School of Medicine, University of Zagreb, Croatia. The provision was approved by the Ethics Committee of the University of Zagreb Medical School (no.: 04-1130-2006). According to Articles 232 (Okon et al., 1980), 235 (Chang, 2002), and 236 (Chang, 2002) of the Law on Healthcare (Republic of Croatia), written consent from patients was not necessary.

Adult human paraffin-embedded testicular tissue (n = 3) was provided by the Department of Urology, University Hospital Zagreb, Croatia. Samples were obtained via testicular biopsy from men diagnosed with obstructive azoospermia (OA) with preserved tubular spermatogenesis and interstitial tissue. Each patient provided written consent for the procedure and the histological analysis/current study. Approval was granted by the Ethics Committee of the School of Medicine at the University of Zagreb (380-59-10106-20-111/171).

### 2.2 Immunofluorescence and imaging

Immunofluorescent (IF) staining was performed on human adult and fetal FFPE (formalin-fixed paraffin-embedded) testis sections (4 µm thickness). Slides were incubated at 55°C, deparaffinized in xylol, rehydrated in 100%, 96%, and 70% EtOH, and then washed in distilled H_2_O. Heat-induced antigen retrieval was performed using a pressure cooker and the Tris-EDTA buffer (10 mM Tris Base, 1 mM EDTA Solution, 0.05% Tween 20, pH 9.0) for 60 min, followed by 30 min of cooling at room temperature (RT). The slides were then washed in PBS (phosphate-buffered saline) 1x buffer. Non-specific binding was blocked by incubating the slides in 2.5% normal horse serum for 20 min. Primary antibodies were prepared in PBS 1x (13.7 mM NaCl, 0.27 mM KCl, 0.65 mM Na_2_HPO_4_, 0.14 mM KH_2_PO_4_, Kemika, HR) containing 0.1% Triton X and 1% normal horse serum in the following dilutions: rabbit polyclonal anti-HSD17B3 antibody 1:500 (13415-1-AP, Proteintech, United States), mouse monoclonal anti-CYP17A1 antibody 1:1000 (sc-374244, Santa Cruz Biotechnology, United States), mouse monoclonal anti-SOX9 antibody 1:100 (AMAb90795, Atlas Antibodies, Sweden). The anti-HSD17B3 antibody was verified in a previous study (Flippo et al., 2022). All samples were double-labeled with anti-HSD17B3 and anti-SOX9 or with anti-HSD17B3: anti-CYP17A and incubated in a humid chamber at 4°C overnight (ON). Testicular tissue with complete spermatogenesis from men with OA was used as positive controls, and samples on which primary antibodies weren’t used were included as negative controls. The next day, slides were washed twice in PBS 1x for 10 min. Slides were also incubated with an amplifier anti-rabbit antibody for 15 min. Slides were then incubated with secondary antibodies, donkey anti-mouse (Alexa Fluor 488; ab150109, Abcam, United Kingdom) diluted 1:200 and horse anti-rabbit antibody (VectaFluor™ Excel Amplified Anti-Rabbit IgG, DyLight™ 594 Antibody Kit, Vector Laboratories, United States) diluted in PBS 1x for 2 h at RT in a dark humid chamber. Next, samples were washed 3 × 10 min in PBS 1x and incubated with TrueBlack^®^ lipofuscin autofluorescence quencher solution (Biotium, United States) for 30 s, freshly diluted 20x with 70% EtOH, to reduce the immunofluorescence background. Samples were counterstained with the Hoechst solution (1 ng/mL) for 10 min, air dried for 3 min, covered with mounting medium (Vectashield, Vector Laboratories, United States), and stored at 4°C overnight. Confocal microscopy images were acquired using an Olympus FV1000 microscope with the FV10-ASW software with filters (Olympus, Japan). Images were processed using ImageJ.

### 2.3 Colocalization analysis

Signal colocalization was conducted in the ImageJ software using the EZcolocalization plugin (Stauffer et al., 2018). Colocalization was quantified by calculating the threshold overlap score (TOS), which measures the overlap in signals above threshold intensity values (Sheng et al., 2016). Thresholds were determined automatically using the Otsu algorithm. The TOS was calculated for HSD17B3 and CYP17A1 since both proteins are cytoplasmic.

### 2.4 Signal intensity quantification

HSD17B3 signal intensity was quantified with the ImageJ software by calculating the integrated density and mean gray value of ROIs (Regions of Interest) determined by the automated thresholding algorithm Moments. Integrated density is the sum of pixel values in the selection. The mean gray value is the sum of the values of all the pixels in the selection divided by the number of pixels.

### 2.5 Statistical analysis

Results were statistically analyzed using GraphPad Prism v7.00 Software (California, United States). Differences in quantitative variables were determined by the Student’s t-test and one-way ANOVA. Results were considered statistically significant if *p* < 0.05.

## 3 Results

### 3.1 HSD17B3 is expressed in Sertoli cells of the human fetal testis

**FIGURE 1:**
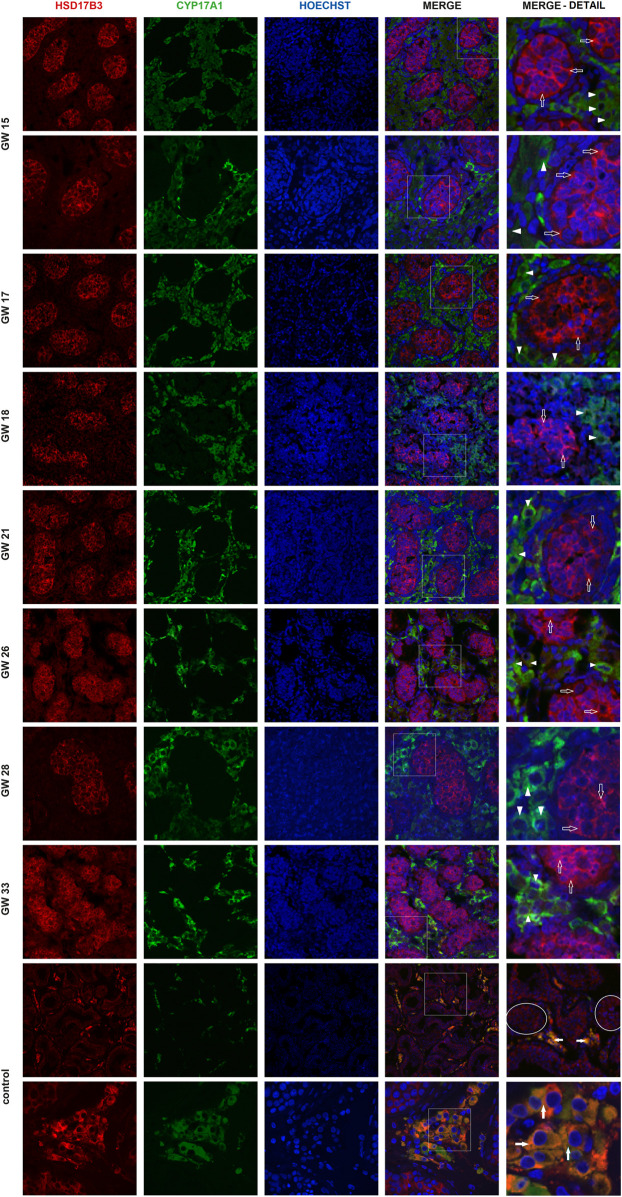
Colocalization of HSD17B3 and CYP17A1 in the human fetal testis. Representative images of immunofluorescent staining showing HSD17B3 and CYP17A1 expression in human fetal testes from gestational week (GW) 15 to GW 33 and in controls, the adult testis. The images show the expression of HSD17B3 in Sertoli cells cytoplasm in the tubules of fetal testes (empty arrows) and their noncolocalization with CYP17A1, expressed in Leydig cells (arrowheads). In adult testis (control), HSD17B3 and CYP17A1 show almost complete colocalization (filled arrows) in adult Leydig cell cytoplasm. Some seminiferous tubules are encircled. The most right column represents enlarged area of a merged image (marked by square). Images are recorded under ×400, except the second row of GW 15, GW 28 samples, and the second row of controls (×600).

**FIGURE 2:**
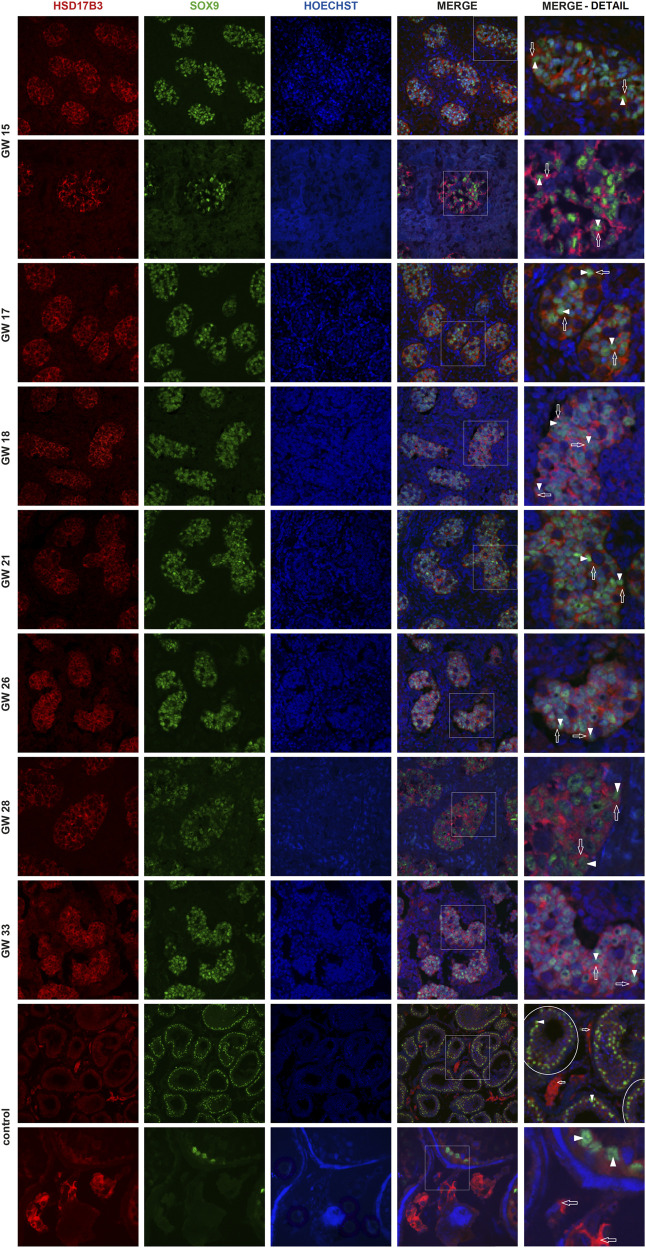
Colocalization of HSD17B3 and SOX9 in the human fetal testis. Representative images of immunofluorescent staining showing HSD17B3 and SOX9 expression in human fetal testes from GW 15 to GW 33 and in controls, the adult testis. The images show the expression of HSD17B3 in Sertoli cell cytoplasm in the tubules of fetal testes (empty arrows), specifically around the nuclei of Sertoli cells, where SOX9 is expressed (arrowheads). In controls, HSD17B3 is expressed in the interstitial Leydig cells, while SOX9 is again observed in Sertoli cell nuclei. Some seminiferous tubules are encircled. The most right column represents enlarged area of a merged image (marked by square). Images are recorded under ×400, except the second row of GW 15, GW 28, and the second row of controls (×600).

Immunofluorescent imaging showed the differential expression of HSD17B3 in human fetal and adult testicular tissue. In the adult testis, HSD17B3 colocalizes with CYP17A1 in the cytoplasm of adult Leydig cells in the interstitial compartment (Figure 1), while the colocalization is not observed with SOX9, a Sertoli cell marker (Figure 2). On the other hand, HSD17B3 clearly shows anti-colocalization with CYP17A1 in the fetal testis (Figure 1). The cytoplasmic HSD17B3 signal is detected around the nuclear SOX9 signal, indicating its presence in the fetal Sertoli cells (Figure 2).

**FIGURE 3:**
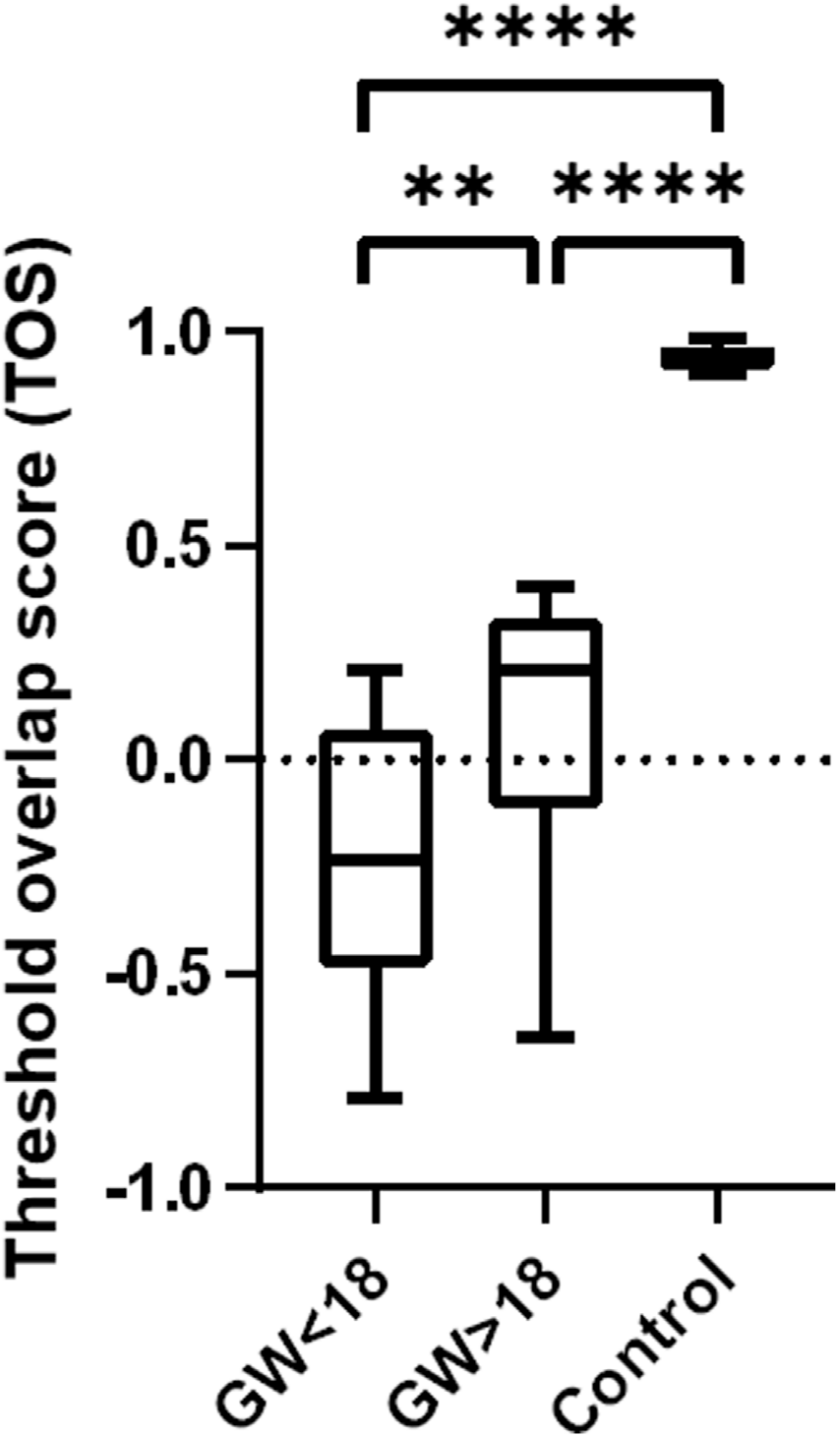
Colocalization analysis of HSD17B3 and CYP17A1 in the human fetal and adult testis (control). Box plot that shows quantification of colocalization through the threshold overlap score (TOS). The TOS points to almost complete colocalization of HSD17B3 and CYP17A1 in the adult human testis, while there is noncolocalization and even weak anticolocalization in the fetal testes. Differences in the TOS are statistically significant between all groups *p < 0.05, **p < 0.01, ***p < 0.001, ****p < 0.0001.

Colocalization was quantified by determining the threshold overlap score (TOS), which measures the overlap in signals above threshold intensity values. The TOS showed almost complete colocalization of CYP17A1 and HSD17B3 in the adult testis. In contrast, the fetal testis showed noncolocalization and weak anticolocalization in the fetal samples before gestational week (GW) 18. Differences in colocalization of CYP17A1 and HSD17B3 were statistically significant between fetal testes before and after GW 18 (*p* < 0.01). Compared with the TOS of the adult testis, which served as a control tissue with known localization of HSD17B3 in Leydig cells, fetal testes before GW 18 and after GW 18 also showed a statistically significant difference. (*p* < 0.0001) (Figure 3). The sample group before GW 18 includes samples from GW 14 to GW 18, while the group after GW 18 includes samples from GW 18 to GW 33.

### 3.2 HSD17B3 expression in the human fetal testis declines during the fetal period

**FIGURE 4:**
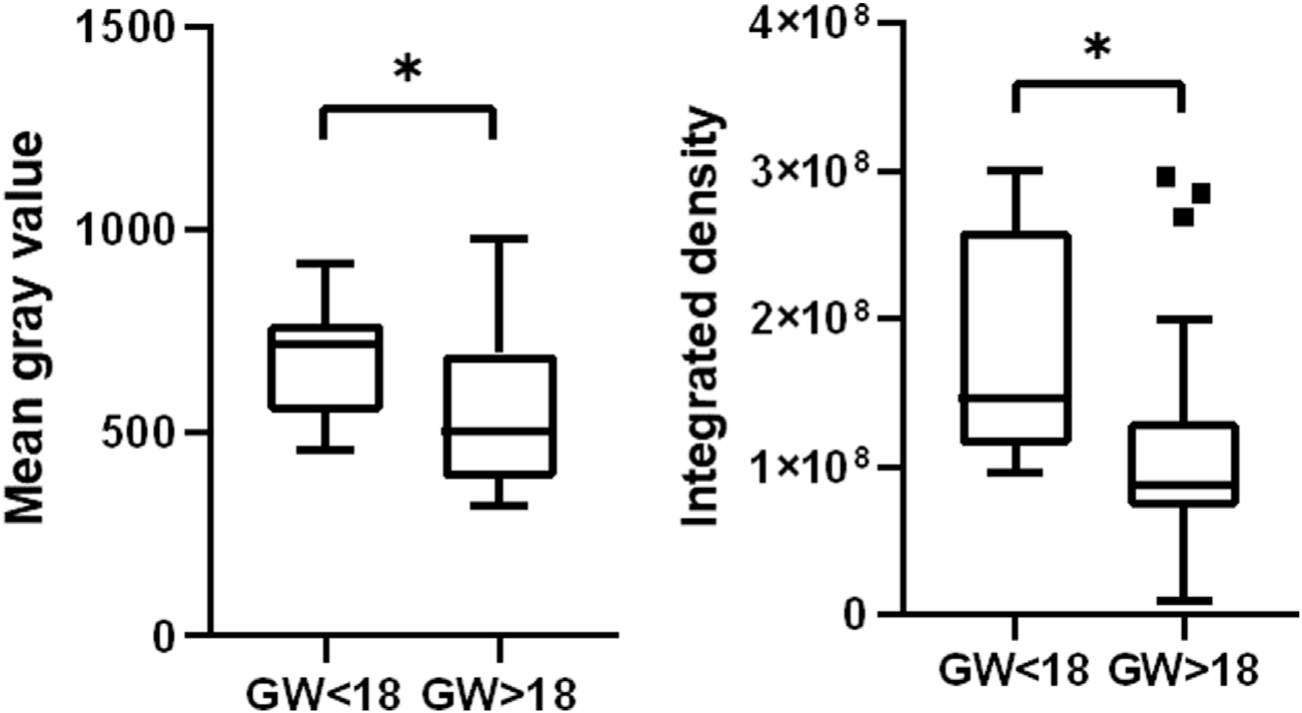
HSD17B3 signal intensity declines during the fetal period. Box plots show higher mean gray value and integrated density in the group before GW 18 than in the group after GW 18 *p < 0.05, **p < 0.01, ***p < 0.001, ****p < 0.0001.

To further investigate the dynamics of HSD17B3 expression in the human fetal testis, we performed a quantitative analysis of HSD17B3 signal intensity across different gestational weeks. Utilizing ImageJ software, we calculated the integrated density and mean gray value of regions of interest (ROIs) identified by the automated thresholding algorithm Moments. The quantitative analysis revealed a statistically significant decrease in HSD17B3 expression as fetal development progresses. Specifically, we observed that the HSD17B3 signal intensity was higher in testicular tissues obtained from fetuses before gestational week 18 (GW 18) than after GW 18. The integrated density values, representing the sum of pixel values within the selected ROIs, indicated a robust presence of HSD17B3 protein in early fetal development, with a marked reduction in later stages. The mean gray value, the average pixel intensity within the selected ROIs, also supported this trend, showing higher values in samples from fetuses before GW 18 (Figure 4). To ensure the reliability of the findings in this study, we performed statistical analyses, and the results demonstrated a statistically significant difference (*p* < 0.05) in HSD17B3 expression, confirming the observed decline in protein levels during the later stages of fetal development.

## 4 Discussion

Using immunofluorescence staining, we demonstrated for the first time that the HSD17B3 protein is highly expressed in the human fetal testis during the second trimester, while expression decreases slightly after GW 18. Hence, this study confirms that the previously observed HSD17B3 mRNA (Guo et al., 2021) is successfully translated in the fetal testis. However, the primary finding of this study is that the HSD17B3 protein is present exclusively in fetal Sertoli cells, while its expression is further switched to Leydig cells in adulthood. Hence, these findings are in accordance with data from the human fetal testis transcriptome (Guo et al., 2021). Finally, they confirm the long-standing hypothesis that human Sertoli cells will likely be the testosterone synthesis’s primary site in the testis.

Moreover, the HSD17B3 antibody could be used as a fetal Sertoli cell marker. While HSD17B3 doesn’t seem to be crucial for basal testosterone production in mice (Rebourcet et al., 2020), HSD17B3 deficiency in humans causes abnormal masculinization, infertility, and very high rates of gender dysphoria, which confirms its role in the development of major sexual characteristics (Longo et al., 1975). Moreover, testosterone levels peak from GW 12 to 18, which overlaps with the masculinization programming window (Tapanainen et al., 1981; O’Shaughnessy and Fowler, 2014) and is reflected in the results of this study, indicating higher HSD17B3 signal intensity in the abovementioned period. Hence, we propose that these disorders in male sexual development could be related to fetal Sertoli cells impairment and not to Leydig cells. This finding also implies a tight, still unresolved, crosstalk between fetal Sertoli and Leydig cells in testosterone production and complex regulation of HSD17B3 expression. In mice, activin A seems to activate the relevant genes in Sertoli cells, but its role in humans still needs to be elucidated (Whiley et al., 2020). HSD17B3 was also found to be highly expressed in adult Sertoli cells in a patient with complete androgen insensitivity syndrome (CAIS), which could be due to a lack of HSD17B3 shift from Sertoli to Leydig cells and could point to a role of androgen signaling in that process (O’Shaughnessy et al., 2012). The elucidation of the hormone regulation switch between Sertoli and Leydig cells could transform the research of novel hormonal treatments.

In this study, we analyzed 14 samples of fetal testicular tissue from GW 14 to GW 33, and thus, we established HSD17B3 protein expression throughout human fetal development for the first time. These samples are rare and difficult to obtain and present a strength of this study. The main limitation of this study is that we analyzed protein expression using immunofluorescence and not Western blot, which is the gold standard, but it was unfortunately not possible to perform since we only had access to paraffin-embedded samples.

This study demonstrated HSD17B3 protein expression in Sertoli cells throughout fetal development and pointed to Sertoli cells as the primary site of fetal testosterone production. Further research is needed to elucidate the background of the HSD17B3 expressional switch and to discover other regulatory players in fetal steroidogenesis.

## Data Availability

The original contributions presented in the study are included in the article/Supplementary Material, further inquiries can be directed to the corresponding author.
